# The *Escherichia Coli* Hfq Protein: An Unattended DNA-Transactions Regulator

**DOI:** 10.3389/fmolb.2016.00036

**Published:** 2016-07-28

**Authors:** Grzegorz M. Cech, Agnieszka Szalewska-Pałasz, Krzysztof Kubiak, Antoine Malabirade, Wilfried Grange, Veronique Arluison, Grzegorz Węgrzyn

**Affiliations:** ^1^Department of Molecular Biology, University of GdańskGdańsk, Poland; ^2^Laboratoire Léon Brillouin, CEA, Centre National de la Recherche Scientifique, Université Paris Saclay, CEA SaclayGif-sur-Yvette, France; ^3^IPCMS/Centre National de la Recherche ScientifiqueStrasbourg, France; ^4^Universite Paris Diderot, UFR Science du VivantParis, France

**Keywords:** Hfq, RNA chaperone, nucleoid associated protein, DNA replication, transposition

## Abstract

The Hfq protein was discovered in *Escherichia coli* as a host factor for bacteriophage Qβ RNA replication. Subsequent studies indicated that Hfq is a pleiotropic regulator of bacterial gene expression. The regulatory role of Hfq is ascribed mainly to its function as an RNA-chaperone, facilitating interactions between bacterial non-coding RNA and its mRNA target. Thus, it modulates mRNA translation and stability. Nevertheless, Hfq is able to interact with DNA as well. Its role in the regulation of DNA-related processes has been demonstrated. In this mini-review, it is discussed how Hfq interacts with DNA and what is the role of this protein in regulation of DNA transactions. Particularly, Hfq has been demonstrated to be involved in the control of ColE1 plasmid DNA replication, transposition, and possibly also transcription. Possible mechanisms of these Hfq-mediated regulations are described and discussed.

## Introduction

The story of the Hfq protein of *Escherichia coli* was initiated in 1968, when the roles of host factors required for replication of bacteriophage Qβ genetic material, which is RNA, have been evidenced (Franze De Fernandez et al., 1972). Subsequent studies demonstrated that there are at least two such factors (Shapiro et al., 1968). One of them was purified and identified as an RNA-binding protein (Franze De Fernandez et al., 1972). It has been named Host Factor I (HF I), as Qβ RNA synthesis *in vitro* by the phage-encoded RNA polymerase was strictly dependent on this protein (Franze De Fernandez et al., 1972). The purified protein was demonstrated to interact with single-stranded RNA, however, no binding of HF I to double-stranded RNA and to single—or double-stranded DNA was detected (Franze De Fernandez et al., 1972). The HF I name has then been replaced with Hfq (for host factor for phage Qβ replication), after cloning and sequencing the corresponding gene (Kajitani and Ishihama, 1991).

Today, the Hfq protein is known to be a major riboregulator that facilitates cellular RNA-RNA interactions. Particularly well documented is the binding of Hfq to small non-coding RNAs (sRNA) that play important roles in the regulation of gene expression at the post-transcriptional level. Enhancement of sRNA-mRNA interaction, which is facilitated by Hfq, most often inhibits translation by blocking the Shine-Dalgarno and/or start codon regions, but also affects RNA stability. This allows bacteria to adapt to their environment, especially in the case of the host infection. The multiple functions of Hfq connected to its interactions with RNA molecules have been recently reviewed in several excellent articles (Vogel and Luisi, 2011; Sobrero and Valverde, 2012; Gottesman and Storz, 2015; Updegrove et al., 2016) In this mini-review, we will focus on other Hfq activities, namely its involvement in DNA transactions.

## Direct interactions between Hfq and DNA

**Figure 1:**
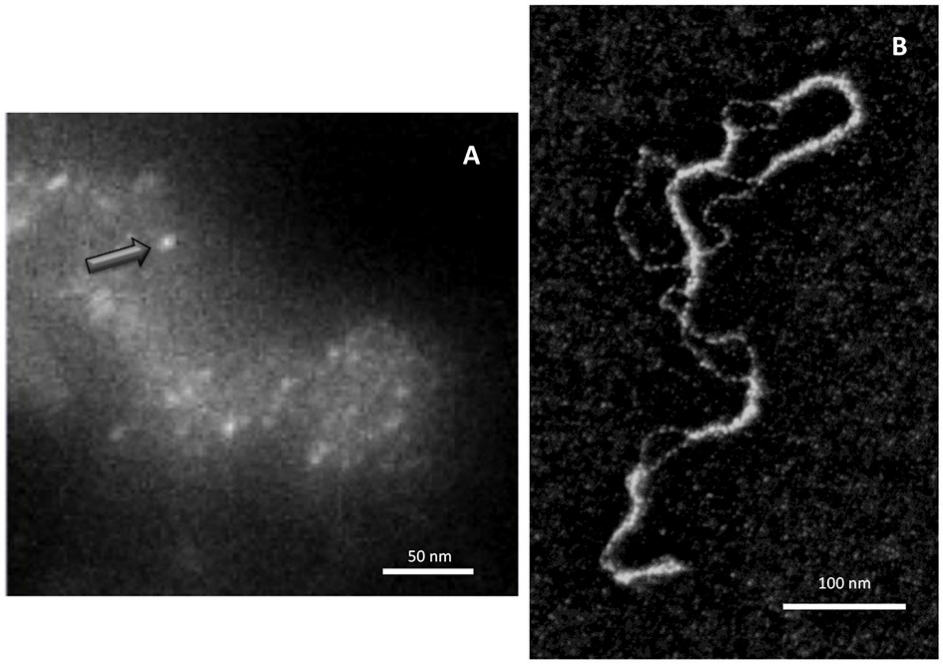
**Direct visualization of Hfq bound to DNA. (A)** *In vivo* analysis. Hfq was labeled with metallothionein (MT) clonable tag for TEM (Diestra et al., 2009) and observed by EELS-STEM (Electron Energy Loss Spectroscopy-Scanning Transmission Electron Microscopy). The image allows to observe and to map gold atoms bound to MT and to observe directly individual Hfq (arrow) along the DNA in the cell (Photo by the courtesy of S. Marco, Institut Curie Orsay). **(B)** *In vitro* analysis. Electron microscopy imaging of Hfq bound to a plasmid dsDNA. After a brief incubation with Hfq, the plasmid is covered and compacted by Hfq (Geinguenaud et al., 2011), showing the potential binding of Hfq to any DNA sequence and its ability to bridge DNA segments (Photo by the courtesy of C. Lavelle and E. Le Cam, IGR Villejuif).

Although early experiments failed to identify Hfq binding to DNA (Franze De Fernandez et al., 1972), the ability of the *hfq* gene product to interact with both supercoiled and linear plasmid DNA has been demonstrated 25 years later (Takada et al., 1997). Clearly, Hfq preferentially binds RNA molecules to DNA: while equilibrium dissociation constants (K_d_) for DNA range from nM to μM (Updegrove et al., 2010; Geinguenaud et al., 2011), for cellular RNA they range from tens of pM for *rpsO* polyadenylated mRNA to nM for sRNAs, such as MicA and DsrA (Folichon et al., 2003; Lease and Woodson, 2004; Fender et al., 2010). For shorter model oligonucleotides, the tightest value measured for oligoriboadenylate (rA_16_) was 1.4 nM, and affinity was 60 times weaker for the corresponding oligodesoxyriboadenylates (dA_6_) *vs.* oligoriboadenylates (rA_6_) (Link et al., 2009). Despite its apparent cellular abundance (10 μM), Hfq low availability *in vivo* questions about its simultaneous binding to RNA and DNA (Hussein and Lim, 2011; Wagner, 2013). Nevertheless, Hfq has been shown to be one of the nucleoid-associated proteins (NAP) (Azam and Ishihama, 1999). If the presence of Hfq in the nucleoid could result from its binding to transcribed RNA, its direct binding to genomic DNA also occurs as DNA fragments are found associated with the purified protein (Updegrove et al., 2010). Note that the direct observation of Hfq in the nucleoid is possible, but difficult taking into account its abundance along the inner bacterial membrane (Azam et al., 2000; Taghbalout et al., 2014). Furthermore, Hfq nucleoid fraction represents 10–20%, while its cytoplasmic and membrane-bound fractions are about 30 and 50%, respectively (Diestra et al., 2009). This makes its observation inside the cell difficult, but its presence along the DNA *in vivo* could be confirmed by electron microscopy imaging of bacteria ultrathin-section (Figure 1A; Diestra et al., 2009).

*In vitro* analyses allowed measuring the K_d_ constants of Hfq-DNA complexes. For the complexes with single stranded dA_20_, the K_d_ was ~ 200 nM, while for double stranded dA_20_-dT_20_ it was ~ 250 nM. This agrees with previous reports indicating that Hfq-bound DNA fragments are of curved topology (Azam and Ishihama, 1999). Moreover, low affinity was measured for dT_20_ and dG_20_ (>1 μM) and no complex was detected for dC_20_ or dC_20_-dG_20_ (Geinguenaud et al., 2011). On the other hand, *in vivo* analyses indicated a preferred Hfq-binding motif (^A^/_T_)T(^A^/_G_)TGCCG (Updegrove et al., 2010). The affinity of Hfq to this motif was slightly lower than to A-tracts. Intriguingly, analysis of identified Hfq-binding sequences indicated that most of them derived from genes coding for membrane proteins (Updegrove et al., 2010). The identified genes did not include any cistrons, which mRNAs were known to be regulated by Hfq, and no previously-described sRNA were encoded by these DNA fragments. This result suggests a general role for Hfq in the regulation of membrane protein expression (Guillier et al., 2006).

Despite a relative high affinity to A-tracts regions, at higher Hfq concentrations the protein interacts with DNA in a sequence-nonspecific manner, as suggested earlier (Takada et al., 1997; Azam and Ishihama, 1999). As seen by molecular imaging, Hfq binds and covers long DNA sequences (Figure 1B). The presence of continuous Hfq stretches, separated by naked DNA, suggests a cooperative binding mechanism by which Hfq could nucleate on high affinity sites followed by spreading along surrounding regions. This model is in agreement with previous observations showing supershifted DNA bands on gel, suggesting multiple Hfq binding to DNA when increasing Hfq concentrations (Azam and Ishihama, 1999; Updegrove et al., 2010).

**Figure 2:**
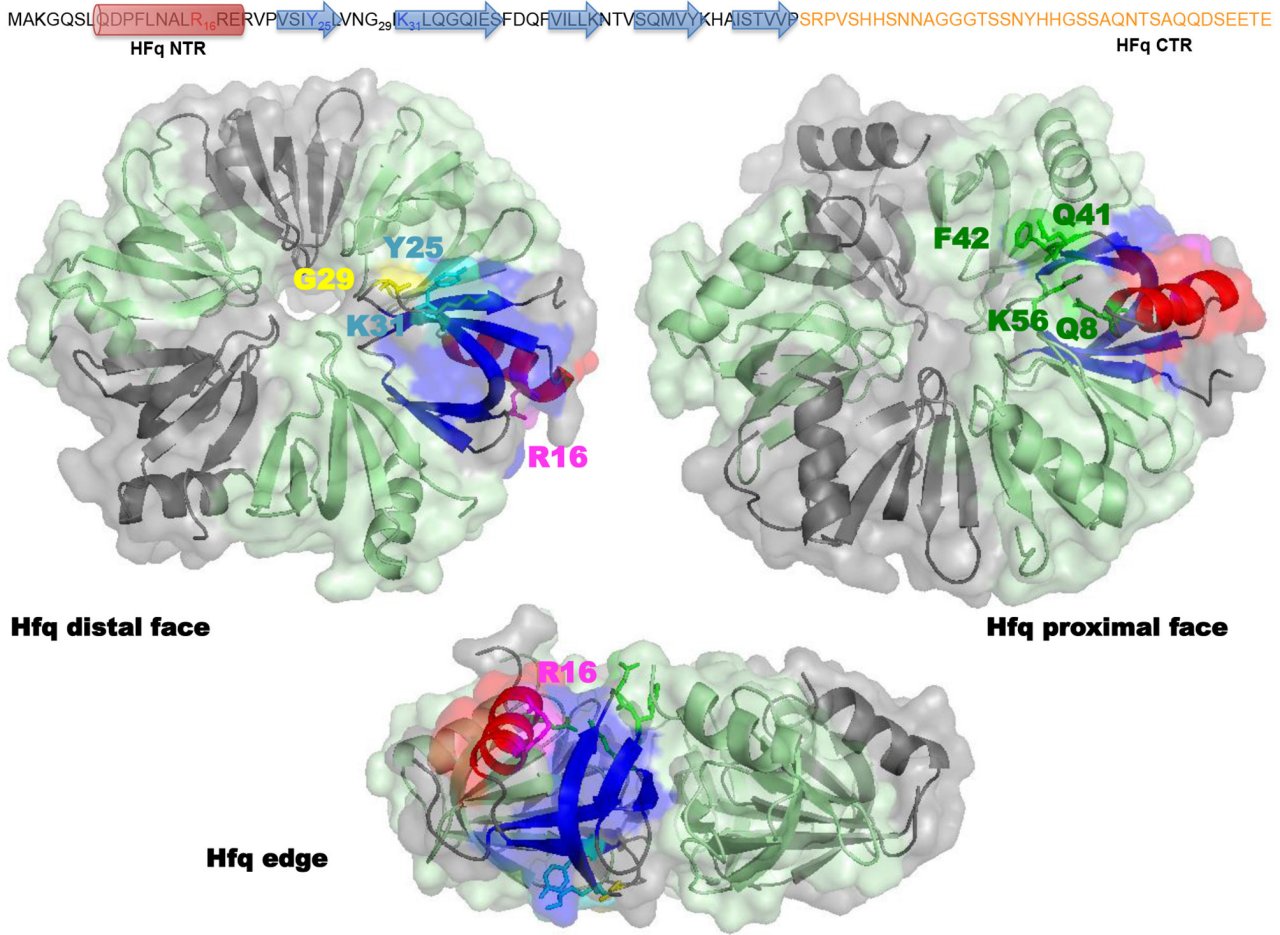
**Molecular representation of Hfq**. Top panel shows the sequence of the full-length *E*. *coli* Hfq: the NTR Sm-fold in black and the intrinsically disordered CTR in orange. Bottom panel shows Hfq NTR structure (PDB accession number 1HK9). To date the CTR has not been visible in high-resolution structures, probably due to its flexibility and/or amyloid behavior. The molecular surface of the hexameric NTR is represented in gray and green. Beta sheets (blue) and alpha helix (red) of the Sm fold are shown as a ribbon in one monomer. In the distal and edge views, stick model evidences Tyr 25, Arg 31 (cyan), Gly 29 (yellow), and Lys 16 (magenta) amino acid residues in this monomer. In the proximal and edge views, stick model evidences Gln 8, Gln 41, Phe 42, and Lys 56 (green) amino acid residues. The CTR (absent in the structure) likely emerges from the edge of the Sm ring.

Structurally, *E. coli* Hfq forms an Sm-fold in its N-terminal region (~ 65 amino acids; Figure 2). This fold consists of a five β-strands antiparallel β-sheet, capped by an α-helix. The β-sheets from six monomers interact with each other to assemble in a toroidal structure with two non-equivalent faces, i.e., the proximal (on which the α-helix is exposed) and distal surfaces (Link et al., 2009; Figure 2). It appears that the distal surface, the edge and the C-terminal region (CTR, ~ 35 amino acids) of Hfq, are involved in DNA binding. Such a conclusion was made on the basis of experiments with *hfq* mutants bearing either deletions of Hfq C-terminal (CTR) domain, or point mutations, such as R16A (edge), K31A and Y25A (distal face) (Updegrove et al., 2010; Figure 2). While CTR is dispensable for RNA binding (Arluison et al., 2004), the distal surface and the edge of the protein seem to be involved in both DNA and RNA binding. Oppositely, the proximal surface seems to be involved in RNA binding only (mainly Q8, Q41, F42, K56, and H57 amino acid residues, Figure 2; Updegrove et al., 2010; Wang et al., 2011). This makes sense taking into account adenylate binding to the distal face, while the proximal side is more dedicated to uridine-rich sequences, absent in DNA (Link et al., 2009; Schu et al., 2015). The preference of Hfq for A-rich RNA over DNA is explained by the formation of a hydrogen bond between the ribosyl 2′ hydroxyl group and the carbonyl oxygen of residue Gly 29 (Link et al., 2009; Figure 2). While dispensing for DNA binding, it was proposed that the C-terminal domain might anchor the Hfq protein to DNA, while the distal face of the Sm-core could be required to direct interactions with the nucleic acid.

Indeed, the involvement of Hfq CTR in DNA binding has also been recently confirmed by low resolution structure of Hfq:DNA complex (Jiang et al., 2015). Previous electron microscopy imaging evidenced that Hfq acts by bridging together two DNA molecules (Figure 1B; Geinguenaud et al., 2011). Such an activity was formerly documented for the H-NS (histone-like nucleoid structuring) NAP. A model for Hfq propensity for bridging has thus been proposed to be linked to its CTR-arm. This interaction results in a change of the mechanical properties of the double helix, and in a compaction of DNA into a condensed form (Jiang et al., 2015). Recently, a new unexpected property of Hfq CTR-arm has also been evidenced. Indeed, the CTR region, which was considered as intrinsically unstructured (Vogel and Luisi, 2011), contains an amyloid sequence that allows the protein to self-assemble (Arluison et al., 2006; Fortas et al., 2015). This new property of the CTR could explain its self-assembly and spreading of Hfq on DNA, but this needs to be investigated further.

Finally, one important question remains about the precise effect of Hfq on DNA *in vivo*. Hfq has been proposed to change DNA superhelicity, precisely plasmids purified in the absence of Hfq are less negatively supercoiled during the stationary phase (Tsui et al., 1994). Nevertheless, this property has never been analyzed in detail. Sugar re-puckering has also been described but should be confirmed by *in vivo* analyses (Geinguenaud et al., 2011).

As for the relative amount of Hfq compared to other NAP, its abundance in actively growing cells is similar to those of the most abundant NAPs, Fis (factor for inversion simulation) or HU (heat-unstable protein). Nevertheless, Hfq quantity in the nucleoid is lower than for HU and Fis, which are present in the nucleoid only. A rough estimation indicate that Hfq represents about 5% of the total nucleoid-associated proteins (vs. 20 and 40% for Fis and HU during the exponential phase of growth, respectively, Talukder and Ishihama, 2015). It is now established that Hfq concentration increases when reaching the stationary phase (Tsui et al., 1997; Diestra et al., 2009; Cech et al., 2014), even if Dps (DNA-binding protein from starved cells) is the main nucleoid-associated protein during this phase of growth. Hfq nucleoid-bound fraction remains more or less constant during the cell cycle (Talukder and Ishihama, 2015). Furthermore, it appears that Hfq can not only interact with DNA, but also: (i) interacts with some NAP, such as H-NS (Kajitani and Ishihama, 1991), (ii) cooperates in the organization of the bacterial chromosome with other proteins, like Fis, HU, H-NS, IHF (integration host factor protein) and StpA (suppressor of td mutant phenotype A; Ohniwa et al., 2013) and (iii) regulates the expression of other NAPs (Lease and Belfort, 2000; Lu et al., 2016). The proteins associated to the nucleoid are usually divided into two groups depending on whether they bridge or bend DNA (Gruber, 2014). While HU, IHF and Fis belong to the bending group of NAP that causes local folding of DNA, H-NS and Hfq belong to the bridging group that organize large parts of chromosomes into isolated domains (Dorman, 2009; Geinguenaud et al., 2011).

Structurally, the nucleoid structure during the exponential growth of *E. coli* is described as a chromatin-like fibers ranging from 5 to 80 nm in diameter (Ohniwa et al., 2013). Atomic force microscopic analyses indicate that over 60% of the fiber structures enter into the "thin" category (i.e., 5–20 nm), and that this chromatin-like structure is significantly condensed upon entering into the stationary phase. The absence of some NAPs can change the relative abundance of “thin” vs. “thick” fibers (Ohniwa et al., 2013). The absence of Hfq results in an increase of both “thin” and “thick” fiber populations. None of the single deletions of NAP were described to cause a significant change in the fraction of the fibers population and more likely the lack of only one nucleoid-associated proteins can be compensated by functions of other proteins from the same group (Ohniwa et al., 2013). Indeed, whether the bridging Hfq and H-NS could replace each other is still unknown.

## Indirect involvement of Hfq in DNA-related processes

The fact that Hfq can interact with DNA and change its properties, inspired studies on the putative role of this protein in various DNA transactions. However, to date, only two processes were investigated in more depth, replication and transposition. Some clues tend to indicate an effect in transcription, but the role of Hfq in this process is still an open question.

### Replication

The role of Hfq in the regulation of DNA replication has been investigated to date only using the models of plasmid replicons. Employing wild-type *E. coli* strain and otherwise isogenic *hfq* mutant, the efficiency of replication of ColE1-like (pMB1—and p15A) and bacteriophage λ-derived plasmids was investigated. In bacteria devoid of Hfq, significant differences in plasmid amount and kinetics of plasmid DNA synthesis were observed relative to wild-type cells, but only for ColE1-like plasmids, not for λ replicons (Cech et al., 2014).

Levels of the Hfq protein in the wild-type strain were increased at the late exponential and early stationary phases of bacterial culture growth relative to the early exponential phase. Accordingly, ColE1-like plasmids replicated in the *hfq* mutant more efficiently than in the wild-type bacteria at late exponential and early stationary phases, while less efficiently at the early exponential phase (Cech et al., 2014). Thus, the differences between wild-type and *hfq* mutant hosts were the most pronounced under conditions corresponding to the highest levels of Hfq in wild-type bacteria. Interestingly, effects of the *hfq* deletion on ColE1-like plasmid replication were impaired in the absence of the *rom* gene (Cech et al., 2014). The regulation of replication of ColE1-like plasmids is based on the action of an anti-sense RNA, called RNA I, which impairs the priming reaction by binding to pre-primer RNA, called RNA II. Since *rom* codes for a protein responsible for enhancing RNA I-RNA II interactions, it was hypothesized that Hfq might either modulate these interactions or interplay with Rom to influence the negative regulation facilitated by this protein (Cech et al., 2014).

In the λ replicons, which are not affected by the absence of Hfq, no RNA-RNA interactions are required for the replication regulation. Although transcription plays a crucial role in the replication initiation from *ori*λ, it appears that Hfq does not modulate the transcriptional activation of this site (Cech et al., 2014).

### Transposition

Hfq influences many cellular processes at the post-transcriptional level mostly by regulating and promoting interactions between RNAs. In addition to direct interactions of Hfq with DNA, indirect effects of the protein on the DNA-related processes have been reported. These include the regulation of transposition process in several known transposon systems in bacteria: Tn10, Tn5, and IS200. In all of these systems, Hfq is a potent inhibitor of transposition as shown in experiments with *hfq*-deficient mutant strains. However, the mode of Hfq action in transposition varies among these systems (Ellis and Haniford, 2016).

For Tn10, Hfq exerts its function typically by promoting base-pairing between transposase RNA (RNA-IN) and its antisense RNA (RNA-OUT) encoded by Tn10/IS10 antisense system (Ross et al., 2013). As a result, downregulation of RNA-IN expression *in vivo* occurs. In addition to this effect, Hfq can inhibit transposase expression independently of the antisense system. It was proposed that Hfq binds directly to RNA-IN, blocking its translation (Ross et al., 2010; Ellis and Haniford, 2016).

Hfq-mediated regulation of transcription of the gene coding for the IS50 transposase might also explain the inhibition of Tn5 transposition (see Transcription; Haniford and Ellis, 2015). Moreover, Crp (cyclic AMP receptor protein) was proposed to mediate Hfq effect on Tn5 transposition (Ross et al., 2014). This, together with the known role of Hfq in stress response processes, supports the hypothesis of linking of transposition to the physiological status of the cell.

IS200 elements, abundant in Enterobacteriaceae, transpose with a very low frequency and are limited by the expression on the transposase gene, *tnpA*. In addition, transposase expression is downregulated by antisense RNA, art200 (Ellis et al., 2015). However, the role of Hfq in the regulation of IS200 transposition does not involve pairing between mRNA and art200. It was shown that Hfq binds upstream of SD sequence of *tnpA* mRNA, inhibiting ribosome binding (Ellis et al., 2015).

### Transcription

Finally, few studies signified a role for Hfq in transcription. So far, how Hfq affects transcription remains unclear. Direct effects could exist as Fourier spectroscopy (FTIR) technique allowed to observe a partial unwinding of a DNA double helix at AT-rich regions by the Hfq protein (Geinguenaud et al., 2011). This opens the discussion on a possible role for this protein in the regulation of transcription initiation. A role for Hfq in the modulation of transcription elongation has also been proposed. According to the hypothesis, interaction of Hfq with a nascent transcript would help to overcome transcription pauses in order to prevent preliminary transcript release (Le Derout et al., 2010). Independently, indirect effects could also occur. Indeed, Hfq function may also be mediated by protein-protein contact and its interaction with RNA polymerase or Rho (a transcription termination factor) have been described (Sukhodolets and Garges, 2003; Rabhi et al., 2011). Transcriptional control by Hfq still remains largely unexplored.

## Conclusions

Although the Hfq protein has been discovered as an RNA-interacting factor, and investigated in this light for many years, it also binds DNA and affects significantly the structure of this nucleic acid. Experimental evidence that Hfq is involved directly or indirectly in different DNA transactions exists, even if molecular mechanisms of these regulatory processes are still poorly understood. Nevertheless, Hfq-mediated control of ColE1-like plasmids appears to be specific, thus, particular mechanisms can be recognized in forthcoming studies. Regulation of transposition and transcription by Hfq might be less complicated than the function of this protein in the control of DNA replication. Regulations at both the post-transcriptional and the DNA-structuring levels allow bacteria to adapt to their environment, with important consequences for its physiology (including virulence). The physiological impacts of Hfq-DNA interactions *in vivo* thus need further investigations.

## Author contributions

GC drafted a part of chapter 2, and chapters 3.1 and 3.3, and participated in preparation of the final manuscript. AS wrote chapter 3.2. KK and AM participated in the general assembly of the text. WG participated in the biophysical description of Hfq. VA drafted chapter 2, prepared figures, and participated in preparation of the final version of the manuscript. GW elaborated the concept of the manuscript, drafted chapters 1 and 4, and prepared the final version of the paper. VA and GW contributed equally to this work.

### Conflict of interest statement

The authors declare that the research was conducted in the absence of any commercial or financial relationships that could be construed as a potential conflict of interest.

## Abbreviations

NAP: Nucleoid Associated Protein
sRNA: Small non-coding RNA
CTR/NTR: C/N-terminal domain.
